# The effect of a phytoestrogen intervention and impact of genetic factors on tumor proliferation markers among Swedish patients with prostate cancer: study protocol for the randomized controlled PRODICA trial

**DOI:** 10.1186/s13063-022-06995-2

**Published:** 2022-12-21

**Authors:** Rebecca Ahlin, Sanna Nybacka, Andreas Josefsson, Johan Stranne, Gunnar Steineck, Maria Hedelin

**Affiliations:** 1 Department of Oncology, Division of Clinical Cancer Epidemiology, Institute of Clinical Sciences, Sahlgrenska Academy at the University of Gothenburg, Box 423, 40530 Gothenburg, Sweden; 2 Department of Molecular and Clinical Medicine, Institute of Medicine, Sahlgrenska Academy, University of Gothenburg, Gothenburg, Sweden; 3 Department of Urology, Sahlgrenska Cancer Center, Institute of Clinical Sciences, Sahlgrenska Academy, University of Gothenburg, Gothenburg, Sweden; 4 Wallenberg Center for Molecular Medicine, Umeå University, Umeå, Sweden; 5 Department of Urology and Andrology, Institute of Surgery and Perioperative Sciences, Umeå University, Umeå, Sweden; 6 Department of Urology, Institute of Clinical Sciences, Sahlgrenska Academy, University of Gothenburg, Gothenburg, Sweden; 7 Department of Urology, Sahlgrenska University Hospital, Region Västra Götaland Gothenburg, Sweden; 8 Regional Cancer Center West, Sahlgrenska University Hospital, Region Västra Götaland Gothenburg, Sweden

**Keywords:** Prostate cancer, Phytoestrogens, Food frequency questionnaire, Gene-diet interaction

## Abstract

**Background:**

A high intake of phytoestrogens, found in soy, rye, and seeds, is associated with a reduced risk of a prostate cancer diagnosis. Previously, we found that the overall decreased risk of prostate cancer diagnosis in males with a high intake of phytoestrogens was strongly modified by a nucleotide sequence variant in the estrogen receptor-beta (ERβ) gene. However, we do not know if phytoestrogens can inhibit the growth of prostate cancer in males with established diseases. If there is an inhibition or a delay, there is reason to believe that different variants of the ERβ gene will modify the effect. Therefore, we designed an intervention study to investigate the effect of the addition of foods high in phytoestrogens and their interaction with the ERβ genotype on prostate tumor proliferation in patients with prostate cancer.

**Method:**

The PRODICA trial is a randomized ongoing intervention study in patients with low- and intermediate-risk prostate cancer with a Gleason score < 8, prostate-specific antigen (PSA) < 20, and scheduled for radical prostatectomy. The study is conducted at Sahlgrenska University Hospital in Gothenburg, Sweden. The intervention consists of a daily intake of soybeans and flaxseeds (~ 200 mg of phytoestrogens) until the surgery, approximately 6 weeks. The aim is to recruit 200 participants. The primary outcome is the difference in the proliferation marker Ki-67 between the intervention and the control groups. The genotype of ERβ will be investigated as an effect-modifying factor. Secondary outcomes include, e.g., concentrations of PSA and steroid hormones in the blood.

**Discussion:**

The results of the PRODICA trial will contribute important information on the relevance of increasing the intake of phytoestrogens in patients with prostate cancer who want to make dietary changes to improve the prognosis of their cancer. If genetic factors turn out to influence the effect of the intervention diet, dietary advice can be given to patients who most likely benefit from it. Dietary interventions are cost-effective, non-invasive, and result in few mild side effects. Lastly, the project will provide basic pathophysiological insights which could be relevant to the development of treatment strategies for patients with prostate cancer.

Trial registration.

ClinicalTrials.gov NCT02759380. Registered on 3 May 2016.

**Supplementary Information:**

The online version contains supplementary material available at 10.1186/s13063-022-06995-2.

## Background

In the clinic, patients diagnosed with prostate cancer often request dietary advice to improve the prognosis of their cancer [1]. We do not know if dietary changes can reduce tumor proliferation in patients with prostate cancer [2]. However, a high intake of phytoestrogens has been associated with a reduced risk of prostate cancer diagnosis [3]. In a previous study, we found an inverse association between dietary intake of phytoestrogens and the risk of prostate cancer, especially among individuals with a particular genetic makeup of the estrogen receptor beta (ERβ) gene [4]. These data warrant a prospective study to investigate whether foods high in phytoestrogens can inhibit the growth of prostate tumors.

Since the 1970s, it has been well known that testosterone and estradiol are involved in the progression of prostate cancer [5]. Phytoestrogens, found in foods such as soy, rye, and seeds [6], are structurally similar to mammal estrogen and bind to ERβ with high affinity [7]. By interacting with ERβ, phytoestrogens could affect prostate cancer progression [8]. Testosterone and its metabolite 5α-dihydrotestosterone (DHT) cause the proliferation of prostate epithelium by binding to the androgen receptor (AR) [9]. In contrast, by binding to ERβ, 5α androstane-3β,17β-diol (3βAdiol), a metabolite of DHT, represses the expression of AR and thereby inhibits androgen-driven proliferation while promoting cell differentiation [10, 11]. In terms of proliferation, current data suggest a combined stimulatory role of estrogen receptor alpha (ERα) and AR in the prostate whereas ERβ inhibits proliferation and stimulates differentiation. Both ERα and ERβ have an affinity for estradiol whereas phytoestrogens and 3βAdiol selectively activate ERβ [10]. Phytoestrogens should, as a result, be able to restrict cancer growth by acting as a substitute for 3βAdiol [12–15].

Several studies have investigated the effect of phytoestrogens on proliferation markers and steroid hormones in patients diagnosed with prostate cancer, but the results have been inconsistent [16–27]. The reason may be that the results have been diluted due to genetic interactions only having effects in some subgroups [4]. Various studies also had few participants, short follow-ups, and varying methods with different doses and sources of phytoestrogens studied [16–27]. The PRODICA (impact of DIet and individual genetic factors on tumor proliferation rate in males with PROstate CAncer) trial was initiated to investigate the effect of dietary phytoestrogens and their interaction with ERβ genotype on prostate tumor proliferation. Our hypotheses in the study are as follows:

1. In males diagnosed with low- and intermediate-risk prostate cancer, the daily addition of 200 mg of phytoestrogen-rich foods to the diet for 6 weeks reduces prostate tumor proliferation compared to no addition of phytoestrogen-rich foods to the diet during the same period.
2. If the effect of phytoestrogens on prostate-tumor proliferation exists, it is modified by males’ polymorphisms in the promoter region of the ERβ gene.

In addition, we will identify the pathways for how a diet high in phytoestrogens may influence the growth of prostate cancer. To determine this, we will identify RNA expression of the ERβ, ERα, and the AR in tumor tissue, as well as steroid hormone concentrations in blood, and compare this between males with a diet high and low in phytoestrogens or between different genotypes of the ERβ gene. We will also evaluate the effect of the intervention diet on phytoestrogen concentrations in the blood depending on the genotype of ERβ.

## Methods and design

The PRODICA study is a randomized controlled dietary intervention trial with patients diagnosed with low- and intermediate-risk prostate cancer, scheduled for radical prostatectomy.

### Study design

#### Recruitment

**Fig. 1:**
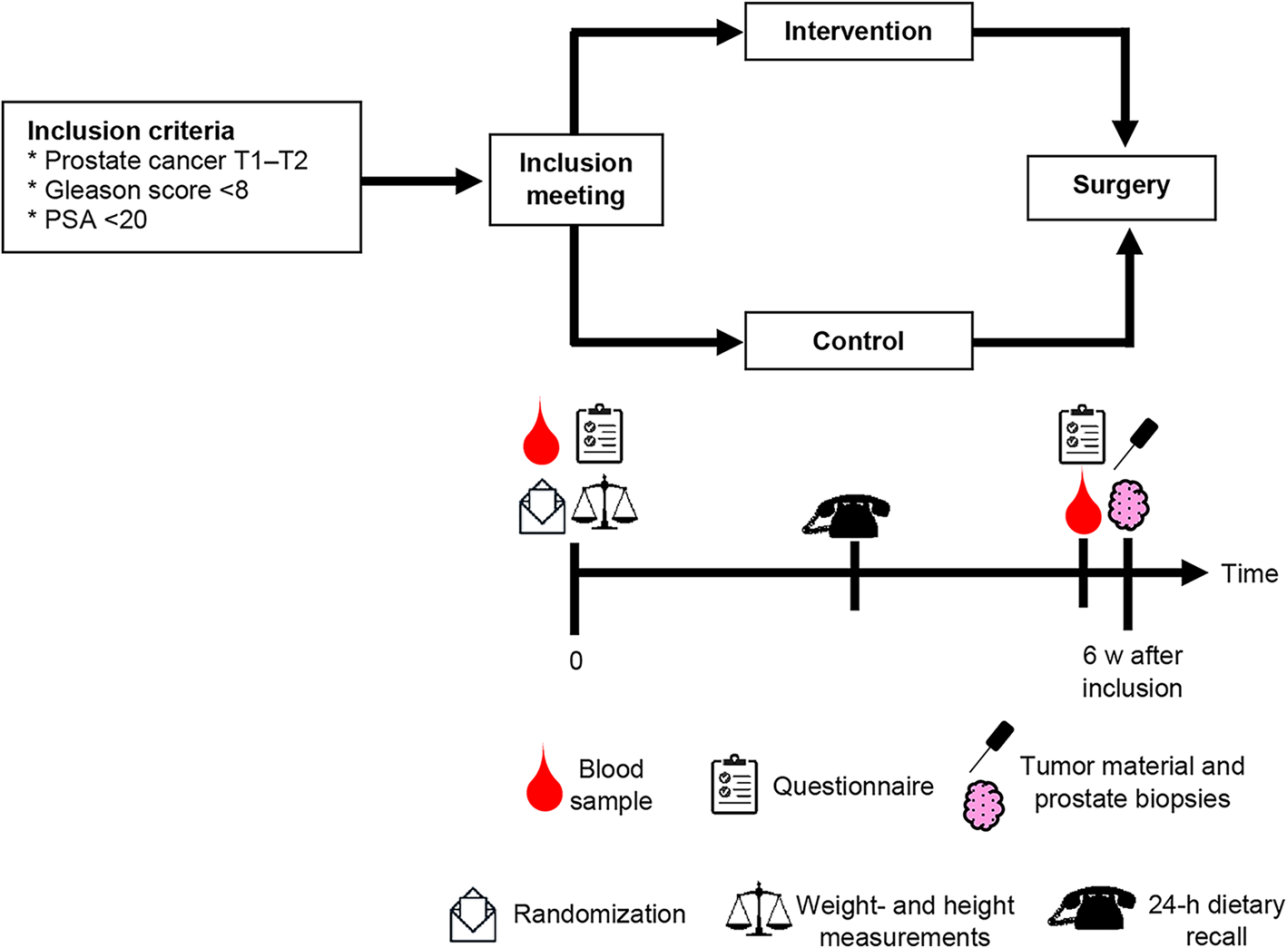
Design of the PRODICA* trial. Eligible patients are identified at Sahlgrenska University Hospital in Gothenburg, Sweden. An inclusion meeting is scheduled with patients who agree to participate. At the inclusion meeting, participants are randomized to an intervention or a control group, they fill out a questionnaire including a food frequency questionnaire, and blood samples are collected. A 24-h dietary recall is performed with participants about halfway through the study to measure compliance with the intervention. Endpoint blood samples are collected, and participants fill out a similar questionnaire again near the time of the surgery. In immediate adjacency to the surgery, biopsies of cancer and benign prostate tissue are sampled for the study, and the prostate is thereafter handled according to clinical routines. *Impact of DIet and individual genetic factors on tumor proliferation rate in males with PROstate Cancer

Participants are recruited at the outpatient urological department at Sahlgrenska University Hospital in Gothenburg, Sweden. The treating physician or the contact nurse asks eligible patients of interest to participate in the study (Fig. 1). The inclusion criteria are patients with prostate cancer T1–T2, Gleason score < 8, prostate-specific antigen (PSA) < 20, and scheduled for radical prostatectomy. The exclusion criteria are ongoing hormone therapy, other difficult physical or psychological conditions or diminished cognitive function, or allergy or intolerance to the intervention foods. A dietitian from the coordinating center contacts the patients that are interested to participate by phone; they will receive initial oral information about the study and can ask questions. An inclusion meeting is scheduled if the patient agrees to participate in the study.

#### Inclusion meeting, randomization, and dietary intervention

At the inclusion meeting with the coordinating center dietitian, participants receive additional oral and written information and sign informed consent. Thereafter, participants fill out a questionnaire online or on paper, including a food frequency questionnaire (FFQ) together with questions about, e.g., meal patterns, physical activity, intake of nutritional supplements, tobacco use, use of medications including antibiotics, socio-economic parameters, anthropometric measurements, and family history of prostate cancer [28]. Participants are randomized to an intervention or a control group by drawing 1 of 26 folded notes from an envelope where half of the notes are labeled intervention and the other half control. Drawn notes are put in another envelope and the notes are reused when all notes are drawn. This procedure is done to achieve an equal number of participants in the two groups. No placebo is used in the control group, but participants in the control group are blinded regarding what intervention foods the intervention group is provided with. Besides the intervention foods, the groups are treated equally, and all participants receive standard care.

During the inclusion meeting, baseline blood samples are collected, and body weight and height are measured. Height is measured using a wall-mounted stadiometer to the nearest 0.1 cm. Participants are instructed to remove heavy objects from their pockets. Participants wear indoor clothing; 1 kg is subtracted from the measured weight to account for their clothes. Both body weight and height are measured without shoes.

All participants receive a brochure with dietary recommendations from the Swedish National Food Agency [29], and the dietitian orally goes through the advice with each patient. The intervention group receive, at the inclusion meeting, fresh frozen green soybeans, roasted yellow soybeans, and flaxseeds in amounts that are estimated to last until the surgery. The intervention foods are purchased from ordinary food suppliers. Participants receive a schedule on how to gradually increase the amounts of the intervention foods during the first 9 days, starting on the day of the inclusion meeting (Additional file 1). Thereafter, they are instructed to eat 47 g of green soybeans, 28 g of roasted yellow soybeans, and 28 g of flaxseeds daily. These amounts are estimated to equal approximately 200 mg of phytoestrogens [30, 31]. Participants also receive serving suggestions and recipes for the intervention foods. We intended to use crushed flaxseeds, but during the study, we had to replace these with whole flaxseeds. In 2017, the Swedish Food Agency dissuaded the intake of crushed flaxseeds due to the formation of hydrogen cyanide from cyanogenic glycosides [32, 33]. During the study, participants are not given any dietary restrictions except for instructions to avoid nutritional supplements.

#### Follow-up and endpoint

A 24-h dietary recall is performed by phone with participants about halfway through the study period. During a clinical visit about 1 week before the surgery, or on the day of the surgery, endpoint blood samples are collected. Participants receive instructions to fill out a similar questionnaire as at baseline, 1 to 2 days before the surgery. Participants receive reminders for both the endpoint blood sample and the endpoint questionnaire. At the surgery, the surgeon collects biopsies, and the radical prostatectomy tissue is handled according to clinical routines.

### Evaluation of the protocol and preparatory analyses

We intended that the intervention should last at least 6 weeks. However, as the time to surgery sometimes was shorter than 6 weeks and few patients wanted to postpone their surgery, the inclusion rate became too slow. We, therefore, decided to include all patients with at least 2 weeks to scheduled surgery. We also aimed to have the prostate biopsies to measure the primary outcome and compare it with patients’ diagnostic biopsies. However, to enable measuring different areas of the tumor and avoid missing data, we choose to use prostatectomy specimens to measure our primary outcome.

When ten participants had been included in the study, we evaluated the study protocol and found that all practical arrangements and established collaborations worked satisfactorily. To test compliance, daily dietary intake was measured with the 24-h dietary recall and the questionnaire, among 13 and 12 participants in the intervention and control groups, respectively. We found no differences between the groups in background characteristics or dietary intake at baseline. The intervention group increased their intake of the intervention foods, and thus the intake of phytoestrogens, while the control group did not change their dietary habits during the study period.

### Dietary assessment

#### Food frequency questionnaire

Intake of energy and nutrients has been validated in the FFQ using urine alkylresorcinol metabolites as markers of dietary whole-grain intake and by comparing intakes with 4-day estimated food records in 30 males with prostate cancer and 31 males without prostate cancer [28]. The FFQ includes 184 food items and complex dishes and is divided into twelve food categories. The participants first answer how many times per day, week, or month they consume the food or the dish. Thereafter, they distribute the sum of ten ingredients that the dish could be made of or specific food items included in the food group. The FFQ at baseline reflects the dietary intake during the past 3 months, and the FFQ at endpoint reflects the time during the study. Standard portions together with the content of energy and nutrients are based on information from the Swedish National Food Agency [34]. We are using an in-house developed database to calculate the estimated intake of phytoestrogens; the database is described elsewhere [30, 31]. After participants have completed the questionnaire, the coordinating center dietitian reviews the questionnaire for eventual errors and contacts the study participant if needed.

#### Twenty-four-hour dietary recall

The 24-h dietary recall is performed by telephone according to a template (Additional file 2) by the coordinating center dietician. First, a simple list of yesterday’s food intake is assessed. Second, a list of foods that might be forgotten is checked (Additional file 2). Third, the time of meals and food details including brand, fat content, cooking methods, and estimated quantities are investigated. Finally, the listed foods are read out for the participant to see if something was forgotten. The 24-h dietary recall is performed to promote and control compliance and measure the total intake of phytoestrogens in the groups. During the phone call, participants in the intervention group can report adverse effects, e.g., flatulence and bloating, of the intervention foods; thus, the amounts of the intervention foods can be regulated based on the information given. Any reason for discontinuation of the intervention foods is not a reason for withdrawal from the study.

#### Concentrations of phytoestrogens in blood

In a proportion of participants, plasma concentrations of different phytoestrogens (daidzein, enterodiol, enterolactone, equol, genistein, glycitein, lariciresinol, and secoisolariciresinol) will be analyzed at Aarhus University in Denmark using LC–MS/MS measurements performed on microLC 200 series (Eksigent/AB Sciex, Redwood City, CA, USA) and QTrap 5500 mass spectrometer (AB Sciex, Framingham, MA, USA) [35, 36].

#### Dietary compliance

Compliance is controlled at two time points by the question of remaining intervention foods: during the phone call when the 24-h dietary recall is performed and at the end of the intervention. The total reported intake of phytoestrogens at the 24-h dietary recall will also be calculated in both groups.

### Blood and biopsy collection

Blood samples are collected by ordinary venipuncture according to laboratory routines by experienced healthcare professionals: in serum, lithium-heparin, and EDTA collection tubes. Serum and EDTA collection tubes are stored at room temperature for at least 30 min; thereafter, the serum tubes are centrifuged, and the EDTA tubes are refrigerated. Lithium-heparin tubes are centrifuged within 30 min. The serum and lithium-heparin collection tubes are centrifuged at 2000* g* for 10 min. The samples are pipetted into microtubes, frozen at − 20 °C the same day as the sampling, and stored at − 80 °C before they will be sent for analysis in batches. All samples are stored in the biorepository of “Biobank Väst,” registration number 890.

In immediate adjacency to the surgery, the surgeon collects four median-needle biopsies from the fresh prostate specimen, two from the tumor site and two from healthy prostate tissue, based on the previous results from the diagnostic biopsies and magnetic resonance imaging. The time of sampling and refrigeration is recorded. One of the benign and one of the cancer biopsies are put in RNAlater overnight; the RNAlater is thereafter removed. When the RNAlater has been removed, the biopsies are frozen at − 20 °C and stored at − 80 °C before they will be sent for analysis. The two other extra biopsies are sent for ordinary clinical pathological analysis to confirm that they were obtained from benign and cancer tissue. The radical prostatectomy tissues are formalin-fixed paraffin-embedded according to clinical routine.

### Other collection

Total PSA concentrations at diagnosis and concentrations that are missing at endpoint, Gleason score, tertiary Gleason grade, T-stage, number of prostate biopsies, number of prostate biopsies with cancer, and prostate volume are collected at different time points from the National Prostate Cancer Register of Sweden [37]. The prostate tumor volume will be collected by using the pathological-anatomical information established after the surgery. PSA density will be calculated from total PSA concentrations and prostate volume. Information on the possible use of Finasteride, a 5α-reductase inhibitor, will be collected from medical records.

### Outcome measures

#### Primary outcome

The primary outcome is tumor proliferation measured with Ki-67 in prostatectomy specimens. Ki-67 is a common marker of tumor proliferation, it can predict survival in localized prostate cancer, and it is based on cellular expression during the cell cycle [38]. Ki-67 will be assessed with an immunohistochemical method (CONFIRM anti-Ki-67 [30–9] Rabbit Monoclonal Primary Antibody; 790–4286; Ventana/Roche), which is described elsewhere [39]. ULTRA Cell Conditioning Solution (Ventana) will be used in the processing of the tissue samples as a pretreatment step. UltraView Universal DAB Detection Kit A will be used to detect antibodies, and the staining process is run in Benchmark Ultra (Ventana). A pathologist will evaluate the Ki-67 index by evaluating five different randomly selected areas of the largest and dominating tumor from the formalin-fixed paraffin-embedded radical prostatectomy tissue. At least 100 cells per area and 500 cells per tumor will be evaluated. The Ki-67 index will be the ratio of immunohistochemically positive prostate cancer nuclei divided by the total number of tumor cells evaluated × 100.

#### Secondary outcomes

Secondary outcomes include (1) blood-based changes of total and free PSA and selected hormones (mentioned below), (2) mRNA expression of genes involved in tumor proliferation and ER signal pathways, and (3) protein expression of androgen, ERβ, and ERα receptors in prostate biopsies.

##### PSA and hormones

Concentrations of total and free PSA, testosterone, estradiol, sex hormone-binding globulin (SHBG), and insulin-like growth factor 1 (IGF-1) will be analyzed in the serum at the Department of Clinical Chemistry (Halland Hospital, Halmstad and Varberg, Sweden), and the ratio between free and total PSA along with ratio between testosterone and SHBG is calculated. PSA and SHBG will be analyzed by sandwich assay on a Cobas 8000 analyzer series (Roche). Testosterone and estradiol will be analyzed using electrochemiluminescence immunoassay (ECLIA) on Cobas 8000 analyzer series (Roche), and IGF-1 will be analyzed by using sandwich enzyme-linked immunosorbent assay (ELISA).

##### Genotyping of estrogen receptor beta

In whole blood samples, the single nucleotide polymorphism rs2987983-13,950 T/C, described in detail elsewhere [4], will be genotyped in the ERβ gene using the PCR-based method KASP™ [40]. The genotyping takes place at Umeå University. Participants will be divided into two subgroups, i.e., alleles of TT or TC/CC.

##### Receptors

Receptor expression will be analyzed in the biopsies by using RNeasy plus Universal Mini Kits (QIAGEN) and real-time PCR at the Department of Bioscience and Nutrition, Karolinska Institute. ERα, ERβ, and AR mRNA expression will be determined using TaqMan assay [41, 42].

##### Gene expression

The expression of genes involved in proliferation and ER signal pathways and cell cycle progression (CCP) gene expression will be analyzed in prostatectomy specimens to identify the expression of genes involved in tumorigenesis by whole transcriptomic profiling. Total RNA will be extracted from formalin-fixed paraffin-embedded sections of tumors and benign tissues using the global transcriptome-wide expression array Clariom™ D (Thermo Fisher). For the analysis of the CCP score, gene expression of the genes involved in CCP is based on the Prolaris® gene panel [43] and the Decipher Score [44]. The genes for the ER signal pathways are based on the genes found to be ER-associated in a study by Thakkar et al. [45] in breast cancer and also found to be up- and downregulated in other studies (*ESR1*, *GATA3*, *XBP1*, *NAT1*, *FOXA1*, *IL1R2*, *SLC39A6*, *CALU*, *ID1*, *ICA1*, *PFKP*, *SCUBE2*, *PLAT*, *CDC2*, *S100A6*, *SLPI*, *SLC2A3*).

### Statistical analysis

#### Sample size calculation

Calculation using the primary outcome (Ki-67) for a study group consisting of 118 patients provides an 80% power for a two-sided test with a level of significance of 0.05 and an effect size of 0.5 [21]. Earlier, we found that approximately 42% of the male population is heterozygous or homozygous for the variant allele (TC/CC) of the ERβ promoter region single-nucleotide polymorphism (SNP) and 58% homozygous for the wild-type allele (TT) [4]. We expect the effect of the intervention would decrease the Ki-67 score in the experimental arm with the variant genotype and have no effect among those with the common genotype. Thus, we arrived at a total sample size of 118/58% = 203 patients.

#### Data analysis and statistical methods

In the design and data collection, we have testing of drugs as the template (randomization, placebo, blinding, no attrition, no differential measuring errors, correct analysis); for the deviations from the perfect situation, we are using epidemiological theory [46] for guiding the analyses and interpreting the results. Statistical analyses will be performed following a statistical analysis plan that is set up a priori. The analysis plan is available at *ClinicalTrials.gov*. Interim analyses will be performed by an independent statistician when about half of the participants have been recruited. Thereafter, a data monitoring committee (DMC) is consulted, and the principal investigator decides whether to continue or terminate the study.

Analyses, based on means and standard deviations or medians and interquartile ranges will be provided to describe the baseline characteristics and outcomes. Differences between the intervention and the control groups will be assessed by using independent *t*-tests or the Mann–Whitney *U* test. The distribution of the data will be studied to apply to statistical tests and model fitting, and both *P*-values and 95% confidence intervals (CI) will be produced. If needed, appropriate transformations will be applied to improve symmetry and normality.

The dietary intake reported from the 24-h dietary recall and the questionnaires will be calculated and presented as descriptive data. The analyzed concentrations of phytoestrogens in the blood will be compared to the reported intake of phytoestrogens from the 24-h dietary recall and the questionnaires using linear regression analyses and kappa statistics. The median, mean, and maximum values from the five areas of Ki-67 will be calculated for each participant even if the median values will be used as the primary outcome. In the primary analyses, Ki-67 values will be dichotomized according to the median value in the study population. The difference between PSA concentrations at endpoint and baseline will be calculated, both for total concentrations and the ratio of free and total. In the primary analyses, the difference in PSA concentrations will be dichotomized into decreased/unchanged and increased concentrations. For hormone concentrations, the difference between endpoint and baseline and the ratio between testosterone and estradiol will be calculated. The association between phytoestrogen intake and cancer proliferation will be evaluated by generalized linear models, which will provide estimates of the risk difference (RDs) and corresponding 95% CIs, stratified by ERβ genotypes. Interactions between phytoestrogen intake and ERβ SNPs on proliferation will be evaluated considering additive effect scales. The group of phytoestrogen intake is included as a continuous variable, and each SNP is represented by an indicator variable (variant or not). The interaction will be assessed in a linear odds model by the product term between the covariates representing phytoestrogen intake and SNP genotypes.

Causal factors of the outcome, not equally distributed between the two groups (possible confounding factors), will be added to fitted models. Potential confounders will be based on previous subject matter knowledge and with the help of directed acyclic graphs. Missing data on potential confounding factors will be handled with multiple imputations using chained equations [47]. Subgroup analyses will be performed with compliant participants calculated from the endpoint FFQ and the 24-h dietary recall. In the intervention group, compliance will be considered as ≥ 80% of the recommended intake of 200 mg of phytoestrogens, which is equal to ≥ 160 mg. In the control group, intakes below 160 mg of phytoestrogens will be considered compliant.

## Discussion

The PRODICA trial aims to evaluate if adding foods high in phytoestrogens to the diet can reduce the prostate-proliferation rate in patients with low- and intermediate-risk prostate cancer over a period as short as 6 weeks. Patients with prostate cancer often make dietary changes following their diagnosis [48], and the use of nutritional supplements is also common in patients with prostate cancer [49, 50]. Hence, it can be assumed that these patients are motivated to participate in a diet study and will have high compliance with the intervention.

We chose to use soybeans and flaxseeds as the source of phytoestrogens. Soybeans contain a high amount of isoflavones, and flaxseeds contain a high amount of lignans [51]. Unlike several other dietary sources, soybeans and flaxseeds do not need any preparation before eating, and adding them to the habitual diet does not require any major dietary changes, which could increase compliance with an intervention. In in-depth interviews with randomly selected prostate cancer patients (not included in this study), we investigated their attitudes toward participation in an intervention study eating soybeans and flaxseeds and found that patients were generally positive to eat the intervention foods. A major increased intake of beans could result in adverse effects of gastrointestinal discomfort, e.g., flatulence and bloating [52]. To handle this, we made a schedule for how to gradually increase the intake of the intervention foods during the first days of the intervention. Participants can also report gastrointestinal effects during the phone call when the 24-h dietary recall is performed, which enables individual regulation of the study foods.

We decided to give all participants dietary advice according to Swedish nutritional recommendations to treat the groups as equally as possible. The advice to avoid nutritional supplements is given due to potential confounding effects [53]. We chose a proliferation marker as the primary outcome instead of PSA since PSA could be affected by other factors than tumor proliferation [54]. Two other studies used Ki-67 to measure proliferation and found positive effects on this marker with flaxseed supplementation [21, 55].

A limitation of the PRODICA study is that we had to change from crushed to whole flaxseeds during the study inclusion. This will result in a decreased uptake of lignans due to decreased bioavailability [56]. However, continuing to provide patients with crushed flaxseeds would not have been ethical when the authority is dissuading intake of it. According to the European Food Safety Authority, whole flaxseeds are expected to have low uptake of cyanide after consumption [32]. Another limitation is that we have to shorten our intended 6-week intervention for some patients, due to shorter surgery queues. A longer intervention would have been more optimal to discover the potential effects of the intervention. However, we cannot influence the que of surgery, and it would not have been ethical to prolong patients’ waiting time for surgery without their permission. To date, only four participants (intervention *n* = 2, control *n* = 2) with ≤ 2 weeks inclusion to surgery have been included. To measure outcomes in, e.g., blood samples, we are using standardized analytical methods. These methods may result in measurement errors like non-differential misclassification, which can dilute our results [57].

Strengths of the PRODICA study include the hypothesis-driven design, the substantial number of participants we intend to include, and the randomization. Providing participants with soybeans and flaxseeds simplifies intake and eliminates purchasing costs for participants. Furthermore, even if participants may have low compliance with the intervention or drop out of the study, we still can collect our primary outcome from the prostate which is stored in the pathology clinic. We will only have missing data for the primary outcome for those who have not undergone surgery (approximately 2%), and this will probably not affect the interpretation of our results. Other strengths include our developed in-house phytoestrogen database, which covers the foods that contain phytoestrogens in a regular Swedish diet, and our previous research. This includes an interview study of dietary habits in patients with prostate cancer, a validation study of the FFQ including face-to-face interviews, evaluations of the protocol, and preparatory analyses of compliance.

The results of the PRODICA study will contribute important information on the relevance of increasing the intake of foods high in phytoestrogens in patients with prostate cancer who want to make dietary changes to improve the prognosis of their cancer. If genetic factors turn out to influence the effect of the intervention diet, dietary advice can be given to patients who most likely benefit from it. Dietary interventions are cost-effective, non-invasive, and result in few mild side effects. Moreover, soybeans and flaxseeds are besides phytoestrogens also high in dietary fiber, which has several positive health effects [58]. Lastly, the project will provide basic pathophysiological insights which could be relevant to the development of treatment strategies for patients with prostate cancer.

## Trial status

The recruitment to the pilot study was started in February 2016. Some administrative changes were done to promote study participation and decrease the risk of dropouts, but no major changes were made to the study protocol. The main recruitment was started on 20 May 2016 and is expected to be finalized by the end of 2024. The study was published at *ClinicalTrials.gov* on 3 May 2016. Protocol version: 4, 2022–08-30. If any major amendments to the current protocol will be done, this will be submitted to the Swedish Ethical Review Authority for approval and will then be updated on *ClinicalTrials.gov*.

## Roles and responsibilities

The coordinating center is located in Gothenburg. An inclusion group consists of clinical-based personnel who are responsible to ask patients of interest to participate and report the interested patients to the coordinating center. The coordinating center mainly consists of dietitians and is responsible to contact interested patients, conducting inclusion meetings and 24-h dietary recalls, and handling collected centrifuged blood samples and collected biopsies. The coordinating center also occasionally contacts the inclusion group about the inclusion rate and, if necessary, a meeting is held with the two groups. The blood samples are collected as usual clinical samples according to sampling referrals.

A trial steering committee designed the study, has overall responsibility, and supervises the coordinating center. The trial steering committee decided on the study endpoints in consultation with experienced urological clinicians. A monthly follow-up meeting is held with the trial steering committee and the coordinating center. A surgery group, with surgeons and a responsible surgery nurse, is responsible for the collection of prostate biopsies; collection kits are received from the coordinating center. A data management team consists of the principal investigator, researchers, PhD students, and a statistician. The DMC has an advisory role and consists of three researchers active in urology or oncology, they will take part in how the data collection progresses and be given access to the interim analyses. After this, the group will communicate its advice in writing to the research group. The DMC members are independent of the sponsor and investigators and do not state any competing interests.

## Supplementary Information

**Additional file 1. **Schedule on intake of the intervention foods. A schedule on how the participants should gradually increase the amounts of the intervention foods.

**Additional file 2. **Template 24-h dietary recall. A template of how 24-h dietary recall is performed by the dietitian.

## Data Availability

The data are manually entered, but the ranges will be checked to be plausible. All study-related information will be stored securely at the study site in areas with limited access. The data are coded with an identification number, and the code key is stored separately from the data. Data are handled confidentially and are collected for research purposes only. The coordinating center is unblinded and all caregivers are blinded to which group the participants are allocated to. We do not anticipate any requirement for unblinding. All executors of the analyses will receive coded samples and will be blinded to whether the samples belong to the intervention or the control groups. The data from the questionnaires are stored in a secure server located in the DC13 in the data center park owned by Hetzner GmbH. The principal investigator and the research group at Sahlgrenska Academy, University of Gothenburg, will have access to the final dataset. The study results will be presented in publications of international scientific journals and at national and/or international conferences. In upcoming manuscripts, the Vancouver criteria for authorship will be followed. The datasets analyzed during the current study and statistical code are available from the corresponding author upon reasonable request, as is the full protocol.

## Abbreviations

PRODICA: Impact of DIet and individual genetic factors on tumor proliferation rate in males with PROstate CAncer
ERβ: Estrogen receptor beta
PSA: Prostate-specific antigen
DHT: 5α-Dihydrotestosterone
AR: Androgen receptor
3βAdiol: 5α Androstane-3β,17β-diol
ERα: Estrogen receptor alpha
FFQ: Food frequency questionnaire
SHBG: Sex hormone-binding globulin
IGF-1: Insulin-like growth factor 1
CCP: Cell-cycle progression
DMC: Data monitoring committee
CI: Confidence interval
