# Correction: The effect of a phytoestrogen intervention and impact of genetic factors on tumor proliferation markers among Swedish patients with prostate cancer: study protocol for the randomized controlled PRODICA trial

**DOI:** 10.1186/s13063-023-07219-x

**Published:** 2023-03-13

**Authors:** Rebecca Ahlin, Sanna Nybacka, Andreas Josefsson, Johan Stranne, Gunnar Steineck, Maria Hedelin

**Affiliations:** 1 Department of Oncology, Division of Clinical Cancer Epidemiology, Institute of Clinical Sciences, Sahlgrenska Academy at the University of Gothenburg, Box 423, 40530 Gothenburg, Sweden; 2 Department of Molecular and Clinical Medicine, Institute of Medicine, Sahlgrenska Academy, University of Gothenburg, Gothenburg, Sweden; 3 Department of Urology, Sahlgrenska Cancer Center, Institute of Clinical Sciences, Sahlgrenska Academy, University of Gothenburg, Gothenburg, Sweden; 4 Wallenberg Center for Molecular Medicine, Umeå University, Umeå, Sweden; 5 Department of Urology and Andrology, Institute of Surgery and Perioperative Sciences, Umeå University, Umeå, Sweden; 6 Department of Urology, Institute of Clinical Sciences, Sahlgrenska Academy, University of Gothenburg, Gothenburg, Sweden; 7 Department of Urology, Sahlgrenska University Hospital, Region Västra Götaland, Gothenburg, Sweden; 8 Regional Cancer Center West, Sahlgrenska University Hospital, Region Västra Götaland, Gothenburg, Sweden

**Correction: Trials 23, 1041 (2022)**

**https://doi.org/10.1186/s13063-022-06995-2**

The original publication of this article [1] contained a typo on page 2. The incorrect and correct information is shown below. The original article has been updated.

Incorrect

In males diagnosed with low- and intermediate-risk prostate cancer, the daily addition of 200 mg of phytoestrogen-rich foods to the diet for 6 weeks reduces prostate tumor proliferation compared to no addition of phytoestrogen-rich foods to the diet during the same period.

Correct

In males diagnosed with low- and intermediate-risk prostate cancer, the daily addition of phytoestrogen-rich foods (~200 mg phytoestrogens) to the diet for 6 weeks reduces prostate tumor proliferation compared to no addition of phytoestrogen-rich foods to the diet during the same period.
