# Supplementary material for: The effect of a phytoestrogen intervention and impact of genetic factors on tumor proliferation markers among Swedish patients with prostate cancer: study protocol for the randomized controlled PRODICA trial
Source: Trials. 2022 Dec 21;23:1041. doi: 10.1186/s13063-022-06995-2 (PMC9768998; doi:10.1186/s13063-022-06995-2)
Supplement: Supplementary file 1 — Additional file 1. Schedule on intake of the intervention foods. A schedule on how the participants should gradually increase the amounts of the intervention foods. [file 13063_2022_6995_MOESM1_ESM.pdf]

Additional file 1

Schedule on intake of the intervention foods

	Day 1	Day 2	Day 3	Day 4	Day 5	Day 6	Day 7
W 1	The day you were at the dietitian:<br>- 0.5 tablespoons flaxseeds<br>- 0.5 dl green soybeans<br>- 1 tablespoon roasted soybeans	- 0.5 tablespoons flaxseeds - 0.5 dl green soybeans<br>- 1 tablespoon roasted soybeans	- 1 tablespoon flaxseeds,<br>- 0.5 dl green soybeans<br>- 1.5 tablespoons roasted soybeans	- 1 tablespoon flaxseeds<br>- 0.5 dl green soybeans<br>- 2 tablespoons roasted soybeans	- 1.5 tablespoons flaxseeds<br>- 1 dl green soybeans<br>- 2 tablespoons roasted soybeans	- 1.5 tablespoons flax seeds<br>- 1 dl green soybeans<br>- 2.5 tablespoons roasted soybeans	- 2 tablespoons flax seeds<br>- 1 dl green soybeans<br>- 2.5 tablespoons roasted soybeans
W 2	- 2.5 tablespoons flaxseeds<br>- 1 dl green soybeans<br>- 3 tablespoons roasted soybeans	- 3 tablespoons flaxseeds<br>- 1 dl green soybeans<br>- 3.5 tablespoons roasted soybeans<br>**This is the daily intake for the rest of the time!**	Try to get into a routine! Perhaps flaxseeds for breakfast, green soybeans for dinner, and roasted soybeans as evening snacks?	**Remember to drink extra water! The intestines need it to be able to process the extra fiber.**			The dietitian will call one day, do you have any questions?
W 3		Try making hummus from the green soybeans and put it on crispbread!					
W 4	Now it's halftime! Remaining from:<br>Flax seeds: ___ package<br>Green soybeans: ___ package<br>Roasted soybeans: __package (fill in quantity)			Maybe try a new recipe? Feel free to search the internet for inspiration!			
W 5							
W 6							The last day! Remaining from: Flaxseeds: ___ package green soybeans: ___ package roasted soybeans: __ package (fill in quantity)

Remember! 3 tablespoons flaxseeds (28 grams), 1 dl (47 g) green soybeans, and 3.5 (28 g) tablespoons roasted soybeans a day. Do not forget to drink extra water, the intestine needs it for the extra fiber in the diet. Good luck!
