# Supplementary material for: The effect of a phytoestrogen intervention and impact of genetic factors on tumor proliferation markers among Swedish patients with prostate cancer: study protocol for the randomized controlled PRODICA trial
Source: Trials. 2022 Dec 21;23:1041. doi: 10.1186/s13063-022-06995-2 (PMC9768998; doi:10.1186/s13063-022-06995-2)
Supplement: Supplementary file 2 — Additional file 2. Template 24-h dietary recall. A template of how 24-h dietary recall is performed by the dietitian. [file 13063_2022_6995_MOESM2_ESM.pdf]

Additional file 2

Template 24-h recall

1) Quick overall list – a simple lining of dietary intake

At what time did you wake up yesterday?

When was the first time you did eat or drink anything?

Did you drink anything during the meal?

I will come back to more specific questions.

What did you eat after that?

Next time you ate something, at what time was that?

When did you go to bed?

Did you eat something at nighttime before/after you woke up/went to bed?

2) Potentially forgotten food

Drinks: coffee, tea, soft drinks, milk, juice

Other drinks: beer, wine, drinks

Sweets: cookies, candy, ice cream, buns/pastries

Snacks: crisps, popcorn, nuts

Fruit, vegetables, cheese

Bread, crispbread

Tasting during cooking

3) Time for intake – Remember to ask about activities

4) Details – food brands

- How was it cooked? With skin, fat? – Frozen/raw/canned? – How much?

5) The last step – read the written food and see if something is forgotten

6) Intervention group <u>only</u>

How much do you have left of the soybeans and flaxseeds?
